# Overexpression of centromere-localized IFIT3 impairs mitochondrial function and disrupts cellular proliferation and cell cycle progression in down syndrome

**DOI:** 10.3389/fgene.2026.1904151

**Published:** 2026-10-14

**Authors:** Peng Zhou, Yanna Liu, Caixia Lei, Fanyi Zeng, Jing-bin Yan

**Affiliations:** 1 Shanghai Children’s Hospital, Shanghai Institute of Medical Genetics, Shanghai Jiao Tong University School of Medicine, Shanghai, China; 2 Shanghai Ji Ai Genetics and IVF Institute, Obstetrics and Gynecology Hospital, Fudan University, Shanghai, China; 3 NHC Key Laboratory of Medical Embryogenesis and Developmental Molecular Biology, Shanghai Key Laboratory of Embryo and Reproduction Engineering, Shanghai, China; 4 Department of Histoembryology, Genetics and Development, Shanghai Jiao Tong University School of Medicine, Shanghai, China

**Keywords:** APEX2 proximity labeling, CENPA-proximal proteome, down syndrome, IFIT3, mitochondrial dysfunction, trisomy 21

## Abstract

**Background:**

Down syndrome (DS, trisomy 21) is the most prevalent human autosomal aneuploidy, driven by non-disjunction of chromosome 21 during maternal gametogenesis. Centromere-kinetochore complexes mediate accurate chromosome segregation, and centromeric protein dysregulation is linked to chromosome instability; nevertheless, how perturbations of centromere-proximal proteins drive cellular pathogenesis in DS remain poorly defined.

**Methods:**

We applied CENPA-GFP-APEX2 proximity labeling coupled with LC-MS/MS quantitative proteomics to systematically profile CENPA-proximal proteins in euploid normal amniocytes (NACs) and trisomy 21 DS-derived amniocytes (DSACs). Differentially abundant candidate proteins (DAPs) were biochemically validated by Western blot, followed by bioinformatic enrichment analyses. The top upregulated candidate Interferon-induced protein with tetratricopeptide repeats 3 (IFIT3) was overexpressed in human induced pluripotent stem cells (hiPSCs) to dissect its functional impacts on DS-relevant cellular phenotypes. RNA-seq transcriptome profiling was conducted to uncover signaling and metabolic pathways perturbed by IFIT3 overexpression, and mitochondrial functional assays were performed to validate bioinformatic predictions.

**Results:**

We quantified 999 total CENPA-proximal proteins via label-free proteomics, identifying 32 DAPs in DSACs relative to NACs (19 upregulated, 13 downregulated). Enrichment analyses revealed robust dysregulation of interferon response and ribosomal protein networks in DSACs, with IFIT3 exhibiting the most significant upregulation. Lentiviral IFIT3-OE in pluripotent hiPSCs triggered G1-phase cell cycle arrest, suppressed cell proliferation, and elevated apoptotic rates. Transcriptome-wide RNA-seq demonstrated strong transcriptional repression of oxidative phosphorylation and mitochondrial ATP biogenesis gene sets, accompanied by direct functional deficits including reduced mitochondrial membrane potential and lowered intracellular ATP levels.

**Conclusion:**

This study identifies IFIT3 as a highly dysregulated CENPA-proximal candidate protein in fetal DS amniocytes. Gain-of-function assays in hiPSCs confirm that elevated IFIT3 expression is sufficient to disrupt mitochondrial homeostasis and recapitulate core DS cellular defects. Our work provides a novel proteomic framework linking centromere-proximal signaling to mitochondrial dysfunction in trisomy 21, and highlights IFIT3 as a candidate mediator of DS pathogenesis.

## 1 Introduction

Down syndrome (DS), or trisomy 21, is the most common human chromosomal aneuploidy, occurring in approximately one in 750 live births. It arises primarily from maternal chromosome 21 non-disjunction during oogenesis, leading to heterogeneous clinical features—including intellectual disability, characteristic facial dysmorphology, low-set ears, hypertelorism, nuchal skin redundancy, and limb malformations (Antonarakis et al., 2004). Advanced maternal age and environmental teratogens are the best-established risk factors (Lamb et al., 1997).

Although the genetic basis of trisomy 21 is well defined, how the extra chromosome drives systemic cellular dysfunction remains unclear. Centromeres direct kinetochore assembly and microtubule attachment to ensure faithful chromosome segregation. Their dysfunction disrupts cell cycle progression, proliferation, and genomic stability (Verdaasdonk and Bloom, 2011). Critically, chromosome 21 non-disjunction—the root cause of DS—depends entirely on intact centromere-kinetochore function (Mastrorosa et al., 2026). CENPA, CENPB, and CENPL each regulate mitotic fidelity non-redundantly: CENPA turnover maintains chromosomal integrity (Wang et al., 2021; Kang et al., 2024); CENPB governs basal checkpoint signaling (Luo et al., 2005); and CENPL modulates proliferation and apoptosis via MEK1/2–ERK1/2 (He et al., 2024). Yet, the centromere-proximal proteome in human fetal DS cells remains uncharacterized—a key gap in DS pathogenesis research.

To determine how centromere-proximal proteome disruption drives cellular dysfunction in DS, we performed APEX2 proximity labeling in DS-derived amniocytes (DSACs), a fetal model retaining native trisomy 21 genomics and epigenetics. APEX2 covalently labels proteins within 20 nm of bait proteins in live cells, preserving true interactions and avoiding co-immunoprecipitation artifacts (Lam et al., 2015; Lobingier et al., 2017); it has been validated for centromere proteomics (Paek et al., 2017; James et al., 2019; Gao et al., 2018; Myers et al., 2018). DSACs show transcriptional dysregulation (You et al., 2020) and flavonoid-sensitive oxidative stress (Guedj et al., 2020). We used this system to map the centromeric proteome and identify key mediators of DS cellular pathology.

## 2 Methods

### 2.1 Construction of plasmids

The CENPA-GFP-APEX2 fusion coding sequence was cloned into the lentiviral vector (Genechem, Shanghai, China), which contains a CMV promoter, multiple cloning site (MCS), and SV40-puromycin resistance cassette. The full-length human IFIT3 coding sequence (NM_001031683.4) was inserted into a CMV-driven lentiviral backbone with EF1α-copGFP and T2A-Puro elements to generate the IFIT3 overexpression (IFIT3-OE) lentiviral construct (Genechem, Shanghai, China). An empty-vector lentivirus served as the negative control. All plasmids were Sanger-sequenced before viral packaging to confirm 100% sequence identity to the reference transcript.

### 2.2 Cell culture and lentiviral transfection

Primary DSACs and matched euploid normal amniocytes (NACs) were obtained from pregnant patients undergoing prenatal diagnosis at Shanghai Ji Ai Genetics and IVF Institute (affiliated with Fudan University Obstetrics and Gynecology Hospital). Karyotyping confirmed DSACs as 47, XX, +21 or 47, XY, +21 and NACs as euploid 46, XX or 46, XY. Donors were matched for gestational age, fetal sex, and passage number; all experiments used cells at passage ≤5 to avoid senescence. Cells were cultured in AmnioMAX™-II Complete Medium (#11269016, Gibco, Carlsbad, CA, United States of America) at 37 °C, 5% CO_2_, and 95% humidity, with *mycoplasma* testing by PCR. For lentiviral transfection, amniocytes were seeded at 1 × 10^5^ cells/well in 6-well plates 18–2.24 h before infection. Viral supernatant was added directly to adherent cells; 24 h later, medium was replaced with fresh AmnioMAX™-II containing 6 μg/mL puromycin (#ST551, Beyotime, Shanghai, China). After another 24 h, puromycin medium was refreshed to remove residual virus. All proximity labeling proteomics were performed strictly at 72 h when centromeric GFP signal was maximal and nonspecific background minimal.

Undifferentiated hiPSCs (Nuwacell, Hefei, China) were maintained under feeder-free conditions with minor lab-specific adaptations. For functional assays, hiPSCs were transduced with IFIT3-OE or empty-vector lentivirus (NC). Pluripotency was confirmed by marker staining prior to all phenotypic analyses.

### 2.3 Cell cycle, proliferation, and apoptosis detection

Cell cycle distribution, proliferation and apoptosis were quantified using commercial kits (#C1052, #C0075S, #C1056, Beyotime, Shanghai, China) according to the manufacturer’s instructions. DAPI or Hoechst 33,342 was used for nuclear counterstaining in immunofluorescence. Flow cytometry data were acquired on a BD FACSCanto II; raw dot plots and summary bar graphs were provided in the supplementary figures.

### 2.4 Antibodies

The rabbit anti-IFIT3 and the anti-PSMD4 polyclonal primary antibodies were purchased from Proteintech (#15201-1-AP, #14899-1-AP, Wuhan, China); the anti-biotin (#7075), HRP-conjugated antibody was obtained from Cell Signaling Technology; the rabbit anti-CENPB (#ab25734) polyclonal primary antibody was purchased from Abcam (Cambridge, UK); the rabbit anti-GAPDH (#AF1186) monoclonal antibody and the Cy3-conjugated secondary antibody (#A0516) were purchased from Beyotime (Shanghai, China). The rabbit anti-P21 (#A19094) and the anti-P27 (#A119095) monoclonal antibodies were purchased from ABclonal (Wuhan, China).

### 2.5 Immunofluorescence staining

Cells were seeded on glass coverslips, fixed with 4% paraformaldehyde (30 min, RT), washed three times with Dulbecco’s Phosphate-Buffered Saline (DPBS), then permeabilized and blocked with 0.2% Triton X-100 (#93443, Sigma-Aldrich, St. Louis, MO, United States of America) and QuickBlock™ Blocking Buffer (#P0222, Beyotime, Shanghai, China) (30 min, RT). Primary antibodies in blocking buffer were incubated overnight at 4 °C; fluorescent secondary antibodies (species-matched) were incubated for 1 h at RT. Nuclei were counterstained with DAPI. Images were acquired by an inverted fluorescence microscope.

### 2.6 Biotin-phenol labeling in live cells

Cells were treated with 500 µM biotin-phenol (BP, #SML2135, Sigma-Aldrich, St. Louis, MO, United States of America), added to complete medium and incubated at 37 °C for 30 min. Subsequently, 1 mM H_2_O_2_ was introduced, and the reaction proceeded for 1 min with gentle shaking at RT to ensure a thorough biotinylation reaction. Additionally, control cells were maintained without H_2_O_2_ treatment. The reaction was then terminated by adding pre-cooled quenching solution containing 5 mM trolox (#238813, Sigma-Aldrich, St. Louis, MO, United States of America) and 10 mM sodium ascorbate (#11140, Sigma-Aldrich, St. Louis, MO, United States of America). Cells were thoroughly washed to eliminate any excess biotin-phenol, using a protocol of three washes with the quenching solution followed by two washes with DPBS.

### 2.7 Biotin-labeled protein enrichment

Adherent cells were washed twice with ice-cold DPBS, detached with trypsin (#C0203, Beyotime, Shanghai, China, 5 min, 37 °C), and quenched with complete medium. Cells were pelleted (1,000×*g*, 5 min, 4 °C), supernatant discarded, and pellets lysed in ice-cold RIPA buffer (50 mM Tris-HCl pH 7.5, 150 mM NaCl, 0.125% SDS, 0.125% sodium deoxycholate, 1% Triton X-100) + 1× protease inhibitor (#87786, Thermo Fisher, CA, United States of America). Lysates were incubated on ice for 30 min (with inversion), clarified by centrifugation (13,000×*g*, 10 min, 4 °C), and supernatants collected. Biotinylated proteins were captured overnight at 4 °C using Dynabeads MyOne Streptavidin T1 (#65602, Thermo Fisher, CA, United States of America). Beads were washed sequentially with RIPA (2×), 1 M KCl (1×), 0.1 M Na_2_CO_3_ (1×), 2 M urea in 10 mM Tris-HCl pH 8.0 (1×), and RIPA again (2×). Bound proteins were eluted in 3× loading buffer containing 2 mM biotin and 20 mM DTT, then heated at 95 °C for 10 min.

### 2.8 Western blot (WB) analysis

Eluted proteins were quantified by Bradford assay and analyzed by WB. Biotinylated proteins were eluted by boiling beads with 10 µL of 5× loading buffer. Samples were electrophoresed on 4%–12% gradient SDS-PAGE gels (#LK210, EpiZyme, Shanghai, China). Proteins were transferred to 0.22 µm PVDF membranes and transfer confirmed by Ponceau S (#P0022, Beyotime, Shanghai, China) staining. Membranes were blocked with 5% non-fat milk in TBST (1 h, RT), then incubated with primary antibodies. Chemiluminescent signals were detected using Clarity Western ECL substrate (Bio-Rad, United States of America) and imaged on a ChemiDoc MP system (Bio-Rad, United States of America).

### 2.9 LC-MS/MS and proteomic data analysis

After APEX2 labeling, biotinylated proteins were eluted, quantified by Bradford assay, and digested with trypsin (Zhang et al., 2016). Samples were solubilized in DB lysis buffer (8 M urea, 100 mM TEAB, pH 8.5), incubated at 37 °C for 4 h, then supplemented with trypsin and CaCl_2_ for overnight digestion. Peptides were acidified, centrifuged (12,000×*g*, 5 min), and desalted using C18 solid-phase extraction. Fractionation was performed by basic-pH RPLC (mobile phases: A = 2% acetonitrile, pH 10.0; B = 98% acetonitrile, pH 10.0; column temperature: 45 °C; detection: 214 nm), followed by vacuum drying.

LC-MS/MS analysis used a nanoElute UHPLC coupled to a timsTOF pro2 mass spectrometer (Bruker, Germany). Lyophilized fractions were reconstituted in 0.1% formic acid in water (mobile phase A) and in acetonitrile (mobile phase B), centrifuged (14,000×*g*, 20 min, 4 °C), and injected. Raw data were searched against the human UniProt database (homo_sapiens_uniprot_2022_9_5. fasta) in MaxQuant with precursor mass tolerance of 20 ppm and fragment mass tolerance of 0.05 Da. Carbamidomethylation (Cys) was set as fixed; oxidation (Met) as variable modification. Peptide-spectrum matches (PSMs) and proteins were filtered at 1% false discovery rate (FDR); only PSMs with >99% confidence and proteins supported by ≥ 1 unique peptide were retained.

Protein abundance ratios (DSACs/NACs) were calculated as mean log_2_FC across biological replicates. Statistical significance was assessed by two-tailed unpaired Student’s t-test (*p* < 0.05). To control false discovery from multiple testing, candidate differentially abundant proteins (DAPs) (*n* = 32) were treated as an exploratory set. All analyses were conducted in R (v3.4.3).

Three independent biological replicates (*n* = 3), each from a distinct donor (DSACs or NACs), were analyzed per comparison to capture inter-individual biological variation. DSACs and NACs were processed in parallel and randomized during digestion and LC-MS/MS to minimize batch effects. Proteins detected in only one group were excluded from differential analysis to avoid infinite fold-changes, and only proteins identified with ≥1 unique peptide in ≥2 replicates were retained. Levene’s test assessed variance homogeneity prior to statistical testing. Given the small sample size (*n* = 3 per group), which limits normality test power, we used 1,000 permutations to estimate the null distribution of the t-statistic, relaxing the normality assumption.

Functional enrichment of the 32 DAPs was conducted via Gene Ontology (GO) and Kyoto Encyclopedia of Genes and Genomes (KEGG) over-representation analysis. Protein–protein interactions were predicted using STRING-database (version 11.5; https://string-db.org) (Jones et al., 2014; Huang da et al., 2009; Franceschini et al., 2013).

### 2.10 RNA-seq and transcriptomic data analysis

Total RNA was extracted from amniocytes and hiPSCs (distinct groups) using TRIzol (Invitrogen, Carlsbad, CA, United States of America). RNA integrity was assessed on an Agilent Bioanalyzer 2,100 (Agilent Technologies, Santa Clara, CA, United States of America) and cDNA libraries were prep vectord, quantified with a Qubit fluorometer, and size-distribution verified by Bioanalyzer. Sequencing was performed on an Illumina platform (Illumina, San Diego, CA, United States of America). Raw reads were quality-controlled and aligned to the human RefSeq genome using HISAT2 (v2.0.5). Differentially expressed genes (DEGs) were identified with DESeq2 (FDR-adjusted *p* ≤ 0.05 and |log_2_FC| ≥ 1). Functional enrichment (GO, KEGG) was conducted using clusterProfiler (adjusted *p* < 0.05); Gene Set Enrichment Analysis (GSEA) was run locally (Broad Institute) with significance defined as |NES| > 1, nominal *p* < 0.05, and FDR <0.25. Up- and downregulated genes were selected symmetrically using |log_2_FC| ≥ 1, avoiding directional bias.

### 2.11 Quantitative realtime PCR (RT-qPCR)

Total RNA was extracted from amniocytes and hiPSCs using TRIzol (Invitrogen, Carlsbad, CA, United States of America). RNA concentration and purity were assessed by Nanodrop (Thermo, Waltham, MA, United States of America). cDNA was synthesized using an Invitrogen reverse transcription kit (Invitrogen, Carlsbad, CA, United States of America). qRT–PCR was performed with TaqMan assays; primers (Generay, Shanghai, China) were listed in Supplementary Table S1 mRNA levels were quantified via the 2^−ΔΔCt^ method, normalized to *GAPDH*.

### 2.12 Assessment of mitochondrial membrane potential and cellular ATP levels

iPSCs were seeded in culture flasks and stained with JC-1 at 60%–70% confluence. After aspiration of medium, cells were washed once with PBS, then incubated with 2 mL culture medium containing 1 mL JC-1 dye (S2003S; Beyotime, Shanghai, China) for 20 min at 37 °C. During incubation, 1× JC-1 staining buffer was prepared by diluting the 5× stock (1 mL) in 4 mL distilled water and kept on ice. After staining, supernatant was removed, cells washed three times with cold 1× buffer, and fluorescence imaged immediately using a fluorescence microscope. All experiments were performed in triplicate.

Cellular ATP content was measured using the ATP Assay Kit (S0026; Beyotime, Shanghai, China). Cells were lysed with ATP Detection Lysis Buffer. To each well, 100 μL ATP detection working solution was added and incubated for 5 min at room temperature, followed by addition of 20 μL sample or standard. Luminescence was read to determine ATP concentration. Protein concentration was measured in parallel using the BCA Protein Assay Kit (P0012; Beyotime, Shanghai, China), and ATP levels were normalized to protein.

### 2.13 Statistical analysis

Histograms were generated using GraphPad Prism 9.3.0 (GraphPad Software, San Diego, California). Data were shown as mean ± standard error of the mean (SEM). Protein expression differences were assessed by two-tailed Student’s t-test. All statistical analyses were performed in IBM SPSS Statistics 26 (SPSS Inc., Chicago). Significance was defined as *p* < 0.05.

## 3 Results

### 3.1 Validation of the CENPA-GFP-APEX2 proximity labeling system in primary human amniocytes

To enable spatially restricted biotinylation of CENPA-proximal proteins at centromeres, we generated a lentiviral vector encoding a CENPA-GFP-APEX2 fusion protein under the control of the CMV promoter (Figure 1A). Lentiviral transduction of primary amniocytes yielded strong, punctate GFP fluorescence at centromeres 72 h post-transduction (Figure 1B), confirming efficient delivery and correct localization of the CENPA-GFP-APEX2 fusion protein. We further rigorously validated APEX2 enzymatic activity via streptavidin–HRP WB (Figure 1C), which exhibited minimal non-specific background biotin labeling. High-resolution confocal immunofluorescence co-staining with an anti-CENPB antibody further confirmed the exclusive centromeric localization of the fusion protein (Figure 1D).

**FIGURE 1:**
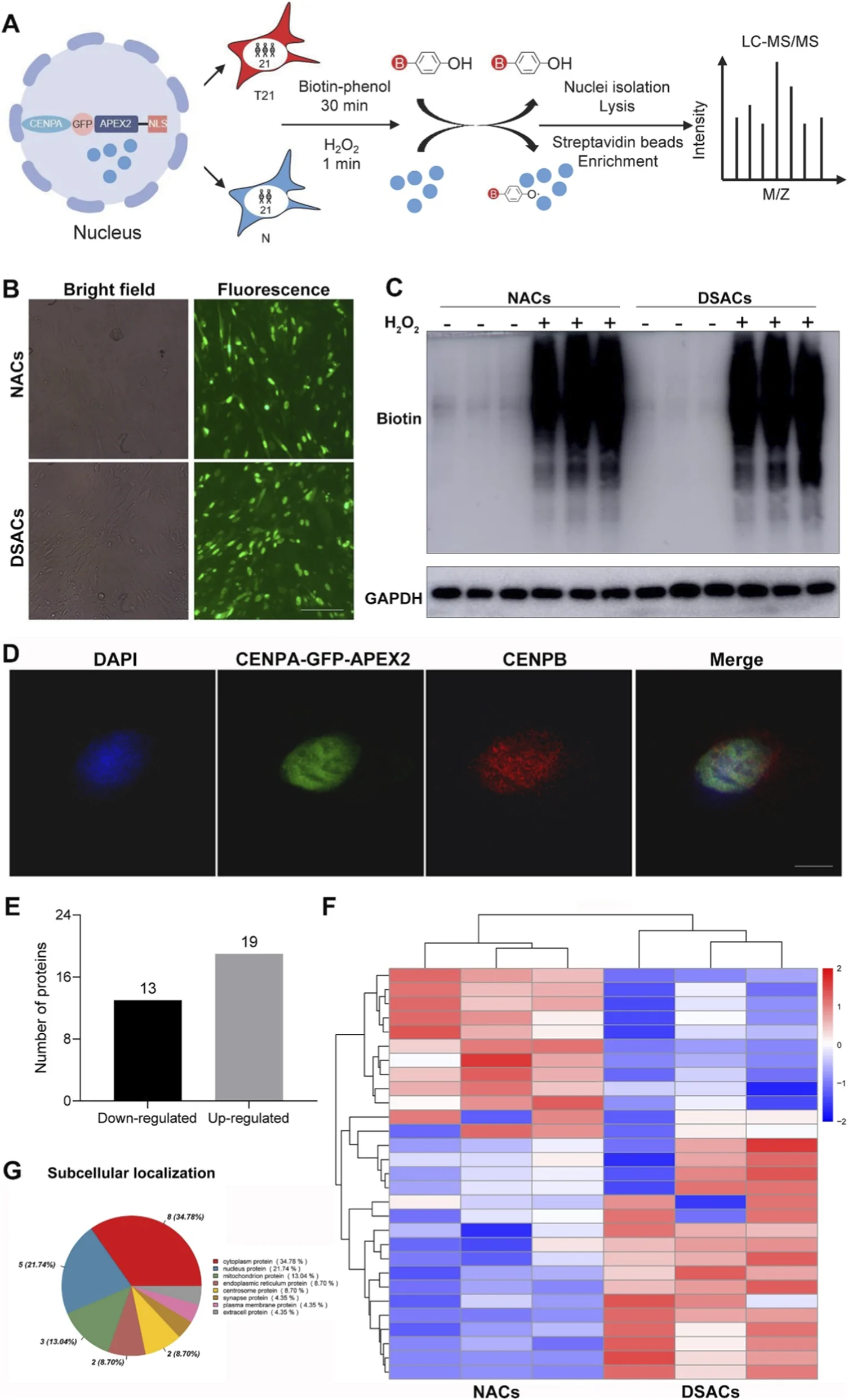
APEX2 proximity biotinylation mapped the centromere-associated proteome **(A)** Schematic of the APEX2-catalyzed biotinylation Workflow **(B)** Bright field and fluorescence images of NACs and DSACs transfected with CENPA-GFP-APEX2 lentivirus (scale bar = 100 µm) **(C)** WB analysis with HRP-conjugated streptavidin to detect biotinylated proteins in cell lysates from cells expressing CENPA-GFP-APEX2 **(D)** Immunofluorescence showing colocalization of CENPA-GFP-APEX2 with the centromeric marker protein CENPB (scale bar = 10 µm) **(E)** A total of 32 differentially abundant proteins (DAPs) were identified, including 19 upregulated and 13 downregulated **(F)** Hierarchical clustering heatmap of DAPs in DSACs and NACs. Expression levels were normalized and hierarchical clustering was performed based on Euclidean distance; red and blue denote upregulated and downregulated proteins, respectively **(G)** Subcellular localization of DAPs.

### 3.2 LC-MS/MS proteomic profiling identifies dysregulated interferon and ribosomal proteins in DSACs

Among the 999 quantifiable CENPA-proximal proteins, we identified 32 significant DAPs: 19 upregulated and 13 downregulated in DSACs (Figure 1E; Supplementary Tables S2, S3). Hierarchical clustering segregated these DAPs into distinct DSACs and NACs expression modules, demonstrating reproducible differential protein abundance across biological replicates (Figure 1F). Subcellular localization annotation revealed that most DAPs resided in the cytoplasm (34.78%) and nucleus (21.74%) (Figure 1G).

Principal component analysis (PCA) of the proteomic dataset revealed tight clustering within each experimental group and clear global separation between DSACs and NACs, indicating stable proteomic profiles within groups and widespread proteome remodeling driven by trisomy 21 (Figure 2A). Volcano plot visualization pinpointed 40 S ribosomal protein S25 (RPS25) as the most strongly upregulated DAP and PSMD4 as the most markedly downregulated DAPs (Figure 2B). We independently validated the expression changes of top hit IFIT3 (upregulated) and PSMD4 (downregulated) via WB using primary amniocytes derived from three donor-matched samples. Consistently with LC-MS/MS quantification, IFIT3 protein abundance was significantly elevated, while PSMD4 levels were reduced in DSACs (Figure 2C). GO enrichment analysis highlighted prominent enrichment in terms related to cellular protein localization, cell ruffles, focal adhesions, COPII vesicle coats, and DNA binding (Figure 2D; Supplementary Table S4). GSEA further confirmed robust upregulation of ribosome biogenesis signatures and repression of RNA recognition motif-containing protein networks (Figure 2E). KEGG pathway enrichment implicated dysregulation of thyroid hormone signaling, platelet activation, influenza A infection, and RAP1 signaling pathways (Figure 2F). STRING analysis identified SUMO2, ACTB, HNRNPAB, and MAPK as core hub proteins within the perturbed centromere-proximal interactome in DSACs (Figure 2G).

**FIGURE 2:**
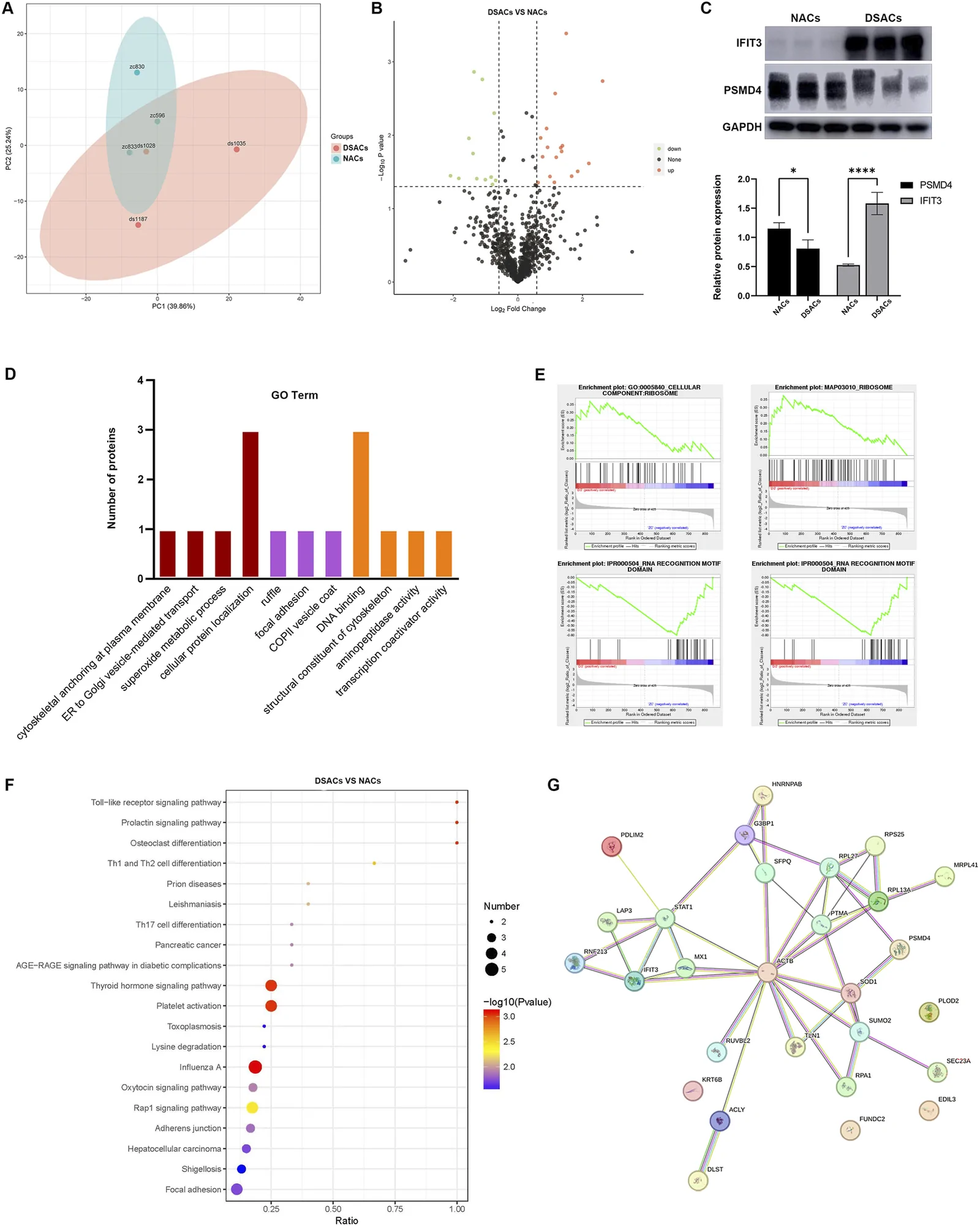
LC-MS/MS profiling of amniocytes derived from fetuses with Down syndrome **(A)** PCA score plot **(B)** Volcano plot of DAPs in DSACs versus NACs. Proteins with |log_2_FC| ≥ 1and adjusted *p* ≤ 0.05 were defined as significantly altered; black dots represent non-significant proteins, red dots denote upregulated DAPs, and green dots denote downregulated DAPs **(C)** WB analysis of IFIT3 (upregulated) and PSMD4 (downregulated), with protein levels normalized to GAPDH as the loading control (*n* = 3; *p < 0.05, ****p < 0.0001, two-tailed Student’s t*-test*) **(D)** GO functional enrichment analysis of the 32 identified DAPs **(E)** GSEA identified dysregulated biological pathways associated with the 32 DAPs **(F)** KEGG pathway enrichment analysis of the 32 DAPs **(G)** STRING-based protein-protein interaction (PPI) network analysis of the 32 DAPs.

Trisomy 21 triggers sustained interferon pathway hyperactivation, leading to global upregulation of IFIT-family interferon-stimulated genes, functions of which have been mechanistically linked to cell cycle arrest and mitochondrial dysfunction (Galbraith et al., 2023; Wu et al., 2025). We selected IFIT3 for downstream functional characterization for three key reasons: it represented the most strongly upregulated DAPs; it is a canonical interferon-stimulated gene with persistent hyperactivation observed across multiple DS cell lines, organoid models, and patient tissue samples; its roles in cell cycle arrest, proliferation suppression, and apoptosis align with core pathological phenotypes of Down syndrome, including impaired neurogenesis, reduced neuronal viability, and growth deficits. Moreover, the detection of IFIT3 within the CENPA-proximal proteome suggests transient mitotic recruitment of IFIT3 to pericentromeric chromatin, consistent with its putative function in safeguarding mitotic fidelity and faithful chromosome segregation.

### 3.3 IFIT3 overexpression in hiPSCs inhibits proliferation, induces G1 arrest, increases apoptosis, and disrupts mitochondrial homeostasis

To dissect cell-autonomous functional defects driven by elevated IFIT3 expression, we generated IFIT3-OE hiPSCs via lentiviral transduction, with empty vector-transduced hiPSCs serving as negative control groups. Consistent green fluorescent signals were detected in both IFIT3-OE and NC hiPSCs under fluorescence microscopy, confirming successful lentiviral transduction (Supplementary Figure S1A). Elevated IFIT3 expression in IFIT3-OE hiPSCs was validated at both mRNA and protein levels (Supplementary Figure S1B). Prior to all functional assays, we confirmed intact pluripotency of edited cells: expression levels of pluripotency markers OCT4 and NANOG were comparable to control fibroblasts (Supplementary Figure S1C), and membrane/cytoplasmic localization of SSEA4 and SOX2 remained unaltered (Supplementary Figure S1D). These results demonstrate that IFIT3 overexpression does not compromise pluripotency or trigger spontaneous differentiation, validating the reliability of subsequent functional phenotyping.

IFIT3 was significantly upregulated in both primary DSACs and IFIT3-OE hiPSCs, accompanied by a marked reduction in cellular proliferation (Figures 3A,B). WB analysis further revealed that P21 and P27, two master negative regulators of cell cycle progression, were substantially downregulated in DSACs (Figure 3C). Cell cycle profiling demonstrated an increased proportion of DSACs entering S phase relative to NACs (Figure 3D), whereas IFIT3-OE hiPSCs exhibited prominent G1-phase cell cycle arrest (Figure 3E). Propidium iodide (PI) staining confirmed a significantly higher apoptotic rate in IFIT3-OE compared with NC hiPSCs (Figure 3F). Collectively, these cellular phenotypes indicate that forced IFIT3 overexpression impairs hiPSC viability and recapitulates hallmark cellular abnormalities observed in DS.

**FIGURE 3:**
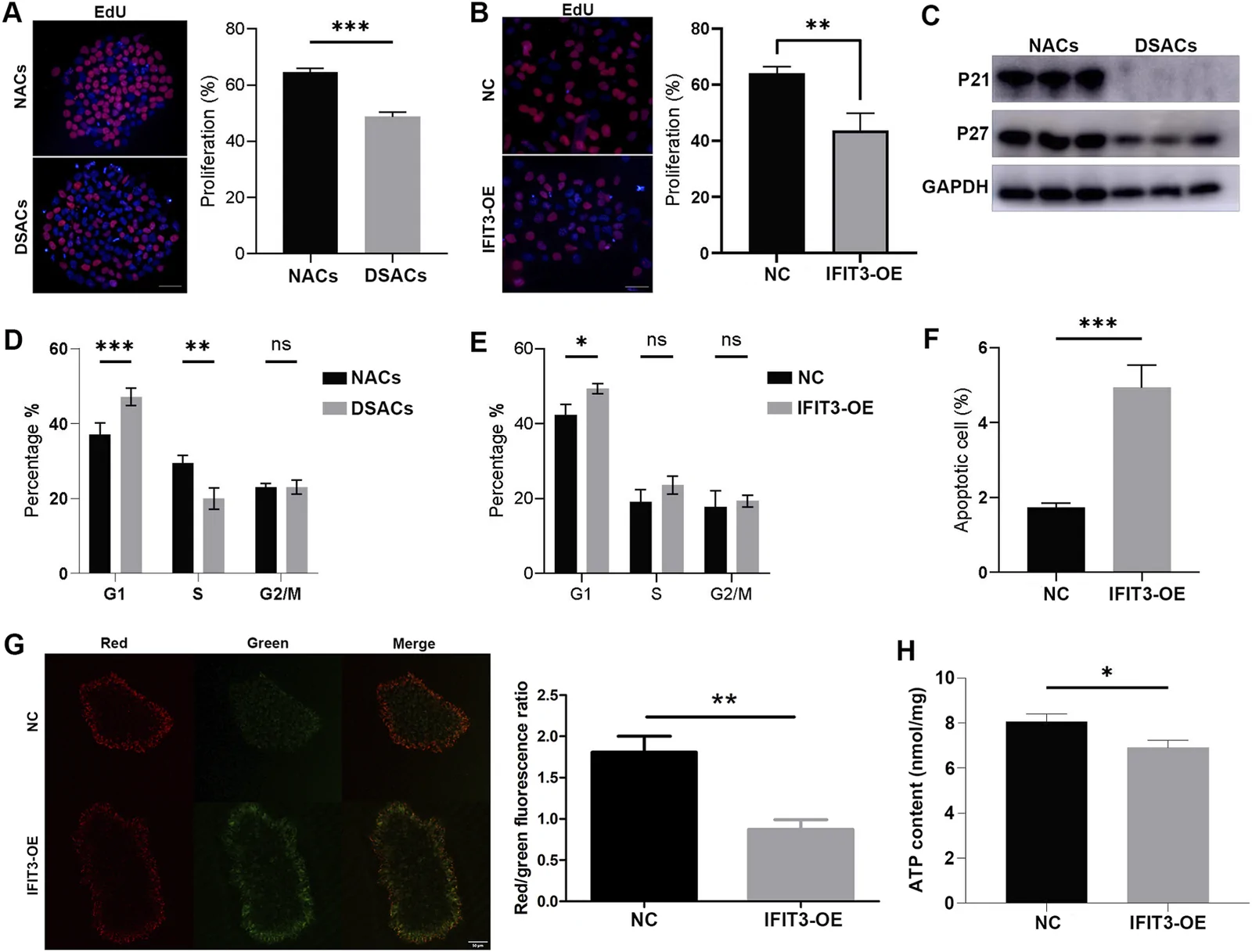
Functional characterization of IFIT3 overexpression **(A)** EdU staining and quantification in DSACs and NACs; scale bar = 50 µm (*n* = 3; ***p < 0.001, two-tailed Student’s t-test) **(B)** EdU staining and quantification in IFIT3-OE and NC hiPSCs; scale bar = 50 µm (*n* = 3; ***p < 0.001, two-tailed Student’s t*-test*) **(C)** WB analysis of P21 and P27 protein expression levels **(D)** Cell cycle distribution analysis of DSACs and NACs (Values are shown as mean ± SD of three independent experiments. One-way analysis of variance (ANOVA) followed by Tukey’s *post hoc* multiple comparison test was used for statistical comparison among multiple groups. **p < 0.01versus control.). **(E)** Cell cycle distribution analysis of IFIT3-OE and NC hiPSCs (Values are shown as mean ± SD of three independent experiments. One-way analysis of variance (ANOVA) followed by Tukey’s *post hoc* multiple comparison test was used for statistical comparison among multiple groups. *p < 0.05versus control.). **(F)** Apoptosis level analysis of IFIT3-OE and NC hiPSCs (*n* = 3; ***p < 0.001, two-tailed Student’s t*-test*) **(G)** Mitochondrial membrane potential (ΔΨm) was quantitatively assessed in IFIT3-OE and NC hiPSCs using JC-1 staining; scale bar = 50 µm **(H)** Intracellular ATP content measurement in IFIT3-OE and NC hiPSCs (*n* = 3; *p < 0.05).

To uncover the transcriptional mechanisms linking IFIT3 overexpression to DS-associated cellular dysfunction, we performed RNA-seq on IFIT3-OE and NC hiPSCs. PCA and pairwise correlation analysis revealed robust transcriptional divergence between the two groups (Supplementary Figure S2B; Supplementary Figure S2A). We identified 200 DEGs defined by adjusted *p* ≤ 0.05 and |log_2_ FC| ≥ 1, including 82 upregulated and 118 downregulated transcripts (Supplementary Figure S2C; Supplementary Table S5). Hierarchical clustering clearly separated transcriptional profiles of IFIT3-OE and NC hiPSCs (Supplementary Figure S2D). GO and KEGG enrichment analyses revealed significant repression of oxidative phosphorylation and mitochondrial ATP synthesis pathways, alongside activation of cell adhesion signaling cascades (Supplementary Figure S2E; Supplementary Table S6). GSEA independently validated suppressed mitochondrial respiration/oxidative phosphorylation signatures and enrichment of axonal guidance and neurogenesis gene sets in IFIT3-OE hiPSCs (Supplementary Figure S2F). Six candidate DEGs (*AMIGO2, GPC6, BCHE, MOGAT3, IL18, SPOCK3*) were further validated via RT-qPCR, showing high concordance with RNA-seq results (Supplementary Figure S2G).

To experimentally verify predicted mitochondrial dysfunction upon IFIT3 overexpression, we measured mitochondrial membrane potential (via JC1 staining) and intracellular ATP abundance (Figures 3G,H). Compared with NC hiPSCs, IFIT3-OE hiPSCs displayed significant reduced red/green fluorescence ratio (*p* < 0.01) and approximately 20% lower intracellular ATP levels. These data confirm that IFIT3 overexpression impairs mitochondrial function.

## 4 Discussion

Down syndrome is predominantly caused by meiotic non-disjunction of chromosome 21. The centromere–kinetochore machinery mediates microtubule–chromosome attachment to ensure error-free mitotic chromosome segregation. Centromere-associated proteins also coordinate cellular proliferation, cell cycle checkpoint activation, and apoptotic signaling, biological processes (Qi et al., 2021; Feng et al., 2022; Lo et al., 2022; Wang et al., 2024) that are consistently perturbed across DS fetal tissues and preclinical DS models. Although centromere dysfunction has been implicated in DS pathogenesis, the centromere-proximal proteome of human fetal DSACs had not been previously characterized, representing a critical knowledge gap in our understanding of the molecular basis of DS.

Herein, we applied CENPA-GFP-APEX2 proximity labeling to systematically map CENPA-interacting proteins in live DSACs and NACs. This proximity biotinylation approach circumvents technical artifacts inherent to conventional co-immunoprecipitation, including disrupted subcellular compartmentalization and loss of weak/transient protein interactions. APEX2 catalyzes biotin labeling of all proteins within a radius of approximately 20 nm around CENPA, simultaneously capturing stable constitutive centromere components and cytoplasmic/nuclear proteins transiently recruited to centromeres during mitosis. The moderate proportion of nuclear DAPs identified herein supports the model whereby cytoplasmic signaling regulators are recruited to pericentromeric chromatin upon mitotic chromosome condensation.

Comparative proteomic profiling uncovered 32 DAPs distinguishing DSACs from NACs, with prominent perturbation of interferon response and ribosomal protein networks. Chronic interferon hyperactivation is a well-established molecular signature shared across all trisomy 21 cell types; our data extend this signature to the centromeric microenvironment. Core interferon effector proteins MX1, STAT1, and IFIT3 were all significantly enriched in DSACs. MX1 and IFIT3 function as canonical antiviral effector molecules downstream of innate immune sensing. Upregulated MX1 drives constitutive antiviral signaling and impairs centromeric dynein–microtubule coupling via its kinesin-like domain and intrinsic GTPase catalytic activity (Zhou et al., 2022; Courtois et al., 2021); MX1 also reduces DS cell viability by triggering AP-1-dependent inflammatory signaling (Guo et al., 2023). In contrast, the functional contribution of IFIT3 to DS pathogenesis remained uncharacterized prior to this study.

IFIT3 is an interferon-stimulated multifunctional protein with established roles in antiviral defense, RNA binding, and translational repression. Accumulating evidence indicates that IFIT3 also modulates cell cycle progression, apoptosis, and proliferative capacity across diverse human cell types (Xiao et al., 2006; Zhang et al., 2023; Hsu et al., 2013), rendering it a high-priority functional candidate despite its predominant steady-state cytoplasmic localization. In hiPSCs, forced IFIT3 overexpression alone recapitulated key DS-associated cellular defects: suppressed proliferation, elevated apoptosis, G1 cell cycle arrest, and impaired mitochondrial bioenergetics. Integrative RNA-seq and cellular functional assays confirmed coordinated transcriptional and functional repression of oxidative phosphorylation, which was a pathway repeatedly linked to neurodevelopmental deficits in human fetal DS cells and Ts65Dn mouse models (Alldred et al., 2021; Bayona-Bafaluy et al., 2021; Tan et al., 2023). Distinct from our previous work demonstrating that chromosome 21-encoded RUNX1 disrupts mitochondrial function through PI3K–Akt signaling (Liu et al., 2023), the present study identifies a genetically independent, interferon-dependent IFIT3 signaling axis that acts upstream of mitochondrial dysfunction in trisomy 21.

Notably, forced overexpression of IFIT3 induced G1-phase cell cycle arrest in hiPSCs, whereas endogenous upregulation of IFIT3 in primary DSACs promoted increased S-phase entry. This apparent discrepancy likely arises from cell-type-specific divergence in cell cycle checkpoint wiring between differentiated primary amniocytes and pluripotent stem cells, highlighting context-dependent mitotic regulatory functions of IFIT3.

Preclinical animal studies have demonstrated that 7,8-dihydroxyflavone ameliorates mitochondrial dysfunction and rescues neurodevelopmental defects in Ts65Dn DS model mice (Valenti et al., 2021). Our work establishes a testable therapeutic hypothesis: pharmacological suppression of IFIT3 activity may restore intact mitochondrial bioenergetics and reverse core DS cellular pathologies, laying a mechanistic foundation for future preclinical mechanistic and therapeutic intervention studies.

## 5 Conclusion

In this study, we identify IFIT3 as a highly dysregulated CENPA-proximal protein in DS amniocytes. IFIT3 overexpression in hiPSCs induces G1 arrest, reduced proliferation, increased apoptosis, and mitochondrial bioenergetic dysfunction, which are key cellular features of DS. Our findings link centromere-proximal interferon signaling to mitochondrial impairment in trisomy 21, advance our understanding of DS pathogenesis, and nominate IFIT3 as a candidate therapeutic target.

## Data Availability

The raw data supporting the conclusions of this article are publicly available in Zenodo at https://zenodo.org/records/21771179 and https://zenodo.org/records/21803772, a general open-access research repository operated by CERN (the European Organization for Nuclear Research).

## Supplementary material

SUPPLEMENTARY FIGURE S1 IFIT3-OE in hiPSCs. **(A)** Bright-field and fluorescence microscopy images of IFIT3-OE and NC hiPSCs (scale bar = 25 μm). **(B)** qPCR and WB analysis of mRNA and protein expression levels in IFIT3 in the IFIT3-OE and NC hiPSCs. (n = 3, **p < 0.01, two-tailed Student’s t-test). **(C)** qPCR analysis of mRNA expression levels of core pluripotency-associated genes, including OCT4 and NANOG, across IFIT3-OE and NC hiPSCs, human dermal fibroblasts, and undifferentiated human embryonic stem cells (hESCs) (n = 3, *p < 0.05, **p < 0.01, ****p < 0.0001, two-tailed Student’s t-test). **(D)** Immunofluorescence Staining for undifferentiated cell markers Sox2 and SSEA4 in IFIT3-OE and NC hiPSCs (scale bar = 50 μm).

SUPPLEMENTARY FIGURE S2 Verification of pluripotency in hiPSCs. **(A)** Correlation analysis of gene expression profiles between IFIT3-OE and NC hiPSCs. **(B)** PCA plot depicting sample distribution across IFIT3-OE and NC hiPSCs. **(C)** Volcano plot illustrating DEGs between the IFIT3-OE and NC hiPSCs, with DEGs defined aslog2FoldChange ≥ 1 and p ≤ 0.05. **(D)** Hierarchical cluster heatmap visualizing the expression levels of DEGs across experimental conditions. **(E)** GO functional enrichment analysis of both up-regulated and down-regulated DEGs, coupled with KEGG pathway enrichment analysis specifically for down-regulated DEGs. **(F)** GSEA identified significantly enriched or depleted biological pathways associated with IFIT3 overexpression. **(G)** Validation of DEGs at the mRNA level by qRT-PCR in in IFIT3-OE and NC hiPSCs (n = 3, **p < 0.01, ***p < 0.001, ****p < 0.0001, two-tailed Student’s t-test).

SUPPLEMENTARY FIGURE S3 Karyotyping analysis in DSACs, NACs, and hiPSCs. **(A)** Karyotyping analysis in DSACs and NACs. **(B)** Karyotyping analysis in IFIT3-OE and NC hiPSCs.

SUPPLEMENTARY FIGURE S4 Original WB images. **(A)** Original WB images in Figure 1C. **(B)** Original WB images in Figure 2C. **(C)** Original WB images in Figure 3C. **(D)** Original WB images in Figure 3C. **(D)** Original WB images in Supplementary Figure S1B.

SUPPLEMENTARY FIGURE S5 Flow cytometry plots of DSACs, NACs, and hiPSCs. **(A)** Flow cytometry plots of DSACs, NACs (n=3). **(B)** Flow cytometry plots of IFIT3-OE and NC hiPSCs (n=6).
