# Supplementary material for: Overexpression of centromere-localized IFIT3 impairs mitochondrial function and disrupts cellular proliferation and cell cycle progression in down syndrome
Source: Front Genet. 2026 Oct 14;17:1904151. doi: 10.3389/fgene.2026.1904151 (PMC13713015; doi:10.3389/fgene.2026.1904151)
Supplement: Supplementary file 1 [file Supplementaryfile1.docx]

**Supplemental Table S1** Primer list

| Name | Species | Forward primer sequence | Reversed primer sequence |
| --- | --- | --- | --- |
| GAPDH | Ho | TTGCCCTCAACGACCACTTT | TGGTCCAGGGGTCTTACTCC |
| IFIT3 | Ho | GCCATTGAGCTGAGTCCTGA | TGCGGAGGACATCTGTTTGG |
| OCT4 | Ho | CTTGCTGCAGAAGTGGGTGGAGGAA | CTGCAGTGTGGGTTTCGGGCA |
| NANOG | Ho | TTTGTGGGCCTGAAGAAAACT | AGGGCTGTCCTGAATAAGCAG |
| AMIGO2 | Ho | CCTGGGAACCTTTTCAGACTG | GCAAACGATACTGGAATCCACT |
| GPC6 | Ho | TGGAAGACAAGTTAAGCCAACAA | TTCTTATGCCTGGACACAAAAGT |
| BCHE | Ho | GTCAGAGGGATGAACTTGACAG | TGAATCGAAGTCTACCAAGAGGT |
| MOGAT3 | Ho | CTTCTACCTCCCGGTCTATCG | GGGACTGAATACAGGGCCTC |
| IL18 | Ho | TCTTCATTGACCAAGGAAATCGG | TCCGGGGTGCATTATCTCTAC |
| SPOCK3 | Ho | TGACCTGCTATTGGACCAGTC | AGAATGCCTTGGTACACTGTTC |

| **Supplemental Table S2** Upregulated proteins in DS compared to normal derived amniocytes. FC: Fold-Change. | | | | | |
| --- | --- | --- | --- | --- | --- |
| **Protein** | **Gene** | **Description** | **Pvalue** | **FC** | **log_2_FC** |
| A0A087WUQ1 | PDLIM2 | PDZ and LIM domain protein 2 | 0.0000 | Inf | Inf |
| Q8IXM3 | MRPL41 | 39S ribosomal protein L41, mitochondrial | 0.0000 | Inf | Inf |
| P62851 | RPS25 | 40S ribosomal protein S25 | 0.0004 | 2.8208 | 1.4961 |
| A0A7P0T9R0 | MX1 | Interferon-induced GTP-binding protein Mx1 | 0.0018 | 6.1476 | 2.6200 |
| A0A0A0MTR7 | RNF213 | E3 ubiquitin-protein ligase RNF213 | 0.0027 | 2.2260 | 1.1545 |
| A0A1S5UZ07 | TLN1 | Talin-1 | 0.0081 | 1.8515 | 0.8887 |
| E4W6B6 | RPL27 | 60S ribosomal protein L27 (Fragment) | 0.0109 | 1.6303 | 0.7052 |
| Q15436 | SEC23A | Protein transport protein Sec23A | 0.0139 | 2.5983 | 1.3776 |
| A0A024R8S3 | SUMO2 | Small ubiquitin-related modifier | 0.0146 | 1.8898 | 0.9182 |
| V9HWC9 | SOD1 | Superoxide dismutase [Cu-Zn] | 0.0148 | 2.5076 | 1.3263 |
| V9HW38 | LAP3 | Cytosol aminopeptidase | 0.0166 | 2.5566 | 1.3542 |
| Q6IBS5 | DLST | Part of 2-oxoglutarate dehydrogenase complex, mitochondrial | 0.0196 | 1.6992 | 0.7649 |
| Q9Y230 | RUVBL2 | RuvB-like 2 | 0.0200 | 2.2783 | 1.1879 |
| A0A7P0T7D6 | IFIT3 | Interferon-induced protein with tetratricopeptide repeats 3 | 0.0242 | 4.5795 | 2.1952 |
| E7ETU9 | PLOD2 | Procollagen-lysine 5-dioxygenase | 0.0310 | 3.6059 | 1.8503 |
| P42224 | STAT1 | Signal transducer and activator of transcription 1-alpha/beta | 0.0361 | 3.1684 | 1.6638 |
| B7Z737 | BCL2L13 | cDNA FLJ52784, highly similar to Bcl-2-like 13 protein | 0.0365 | 1.9900 | 0.9928 |
| A0A7I2V5L3 | RPL13A | 60S ribosomal protein L13a | 0.0439 | 2.1882 | 1.1297 |
| A0A024R1Y2 | ACLY | ATP-citrate synthase | 0.0441 | 1.5405 | 0.6234 |

| **Supplemental Table S3** Downregulated proteins in DS compared to normal derived amniocytes. FC: Fold-Change. | | | | | |
| --- | --- | --- | --- | --- | --- |
| **Protein** | **Gene** | **Description** | **Pvalue** | **FC** | **log_2_FC** |
| H7C0K8 | FUNDC2 | FUN14 domain-containing protein 2 | 0.0000 | -Inf | -Inf |
| A6PVX3 | PSMD4 | 26S proteasome non-ATPase regulatory subunit 4 | 0.0014 | 0.3909 | -1.3551 |
| D6R9P3 | HNRNPAB | Heterogeneous nuclear ribonucleoprotein A/B | 0.0017 | 0.4670 | -1.0986 |
| B4DHN0 | MAPK | Mitogen-activated protein kinase | 0.0050 | 0.6020 | -0.7321 |
| P06454 | PTMA | Prothymosin alpha | 0.0110 | 0.3503 | -1.5133 |
| A0A384NKW7 | CSE1L | Exportin-2 | 0.0177 | 0.3827 | -1.3857 |
| A0A2R8YGF8 | ACTB | Actin, cytoplasmic 1 (Fragment) | 0.0356 | 0.2360 | -2.0828 |
| A0A2R8Y793 | ACTB | Actin, cytoplasmic 1 (Fragment) | 0.0371 | 0.5642 | -0.8258 |
| O43854 | EDIL3 | EGF-like repeat and discoidin I-like domain-containing protein 3 | 0.0387 | 0.2821 | -1.8255 |
| P27694 | RPA1 | Replication protein A 70 kDa DNA-binding subunit | 0.0390 | 0.4146 | -1.2701 |
| Q13283 | G3BP1 | Ras GTPase-activating protein-binding protein 1 | 0.0404 | 0.4985 | -1.0044 |
| Q9BSV4 | SFPQ | SFPQ protein (Fragment) | 0.0412 | 0.6215 | -0.6861 |
| Q6IBA2 | PC4 | Activated RNA polymerase II transcriptional coactivator p15 | 0.0468 | 0.5801 | -0.7856 |
